# Recommendations Following Hospitalization for Acute Exacerbation of COPD—A Consensus Statement of the Polish Respiratory Society

**DOI:** 10.3390/arm94010004

**Published:** 2026-01-04

**Authors:** Adam Jerzy Białas, Adam Barczyk, Iwona Damps-Konstańska, Aleksander Kania, Krzysztof Kuziemski, Justyna Ledwoch, Krystyna Rasławska, Małgorzata Czajkowska-Malinowska

**Affiliations:** 1 Department of Pneumology, Medical University of Lodz, 90-153 Łódź, Poland; 2 Department and Clinic of Pneumology, Faculty of Medical Sciences in Katowice, Medical University of Silesia in Katowice, 40-635 Katowice, Poland; adagne@icloud.com; 3 Department of Pneumonology, Medical University of Gdańsk, 80-210 Gdańsk, Poland; iwona.damps-konstanska@gumed.edu.pl; 4 Department of Pulmonology, Jagiellonian University Medical College, 30-688 Kraków, Poland; aleksanderkania@interia.pl; 5 Department of Pulmonology, School of Public Health, Collegium Medicum, University of Warmia and Mazury, 10-357 Olsztyn, Poland; k.kuziemski@gumed.edu.pl; 6 Section of Guidelines and Recommendations, Polish Society of Family Medicine, 51-141 Wrocław, Poland; justyna.ledwoch@gmail.com; 7 Department of Pulmonary Diseases and Tuberculosis, Independent Public Healthcare Institution, Saint John Paul II Memorial Specialist Hospital of the Ministry of Interior and Administration, 48-340 Głuchołazy, Poland; krysia6613@op.pl; 8 Department of Pulmonary Diseases and Respiratory Failure with the Subunit of Noninvasive Ventilation and the Subunit of Sleep-Related Breathing Disorders, Kuyavian-Pomeranian Pulmonology Center, 85-326 Bydgoszcz, Poland; m.cz-malinowska@ptchp.org

**Keywords:** chronic obstructive pulmonary disease, COPD, hospital discharge, discharge recommendations, COPD exacerbation

## Abstract

**Highlights:**

**What are the main findings?**

- A multidisciplinary team should be involved in developing discharge instructions after hospitalization for a chronic obstructive pulmonary disease (COPD) exacerbation to ensure clear, consistent, and comprehensive documentation.
- Discharge instructions should be reviewed with the patient during hospitalization and supplemented with educational materials provided separately from the discharge summary.
- A personalized action plan for managing future exacerbations should be included.
- The discharge summary must specify the date of the follow-up appointment and contain a prescription for inhaled medications.

**What are the implications of the main findings?**

- This document provides guidance for Polish healthcare professionals on how to create effective discharge instructions after hospitalization for a COPD exacerbation.
- The recommendations will support healthcare administrators in Poland in optimizing the process of preparing discharge summaries for patients hospitalized due to COPD exacerbations.

**Abstract:**

**Introduction:** This document presents recommendations of the Polish Respiratory Society on discharge instructions following hospitalization for an exacerbation of chronic obstructive pulmonary disease (COPD). **Methods:** The Delphi method was applied to achieve consensus among independent experts. **Results:** Fourteen recommendations were formulated. Experts emphasized that discharge summaries require clear graphical and editorial design to ensure readability for both patients and healthcare professionals. The involvement of a multidisciplinary team was recommended to provide coherent and comprehensive documentation. Discharge instructions should be discussed with the patient during hospitalization and supplemented with standardized educational materials provided separately. These materials should cover inhaler technique, smoking cessation, physical activity, pulmonary rehabilitation, and vaccination. For patients with respiratory failure, home oxygen therapy or non-invasive ventilation must be addressed. Discharge recommendations should highlight modifications in baseline COPD treatment and management of comorbidities. A personalized action plan for future exacerbations is essential, and dietary consultation is advised. Finally, discharge summaries should specify follow-up appointments and include prescriptions for inhaled medications. **Conclusions:** The Polish Respiratory Society recommends that discharge instructions be provided to all patients hospitalized for a COPD exacerbation.

## 1. Introduction

According to the current definition, chronic obstructive pulmonary disease (COPD) is *a heterogeneous lung condition characterized by chronic respiratory symptoms (dyspnea, cough, sputum production, and/or exacerbations) due to abnormalities of the airways (bronchitis, bronchiolitis) and/or alveoli (emphysema), that cause persistent, often progressive, airflow obstruction.* Despite being a preventable and treatable condition, COPD remains one of the leading public health challenges worldwide [1].

The estimated global prevalence of COPD among adults aged 40 years and older in 2020 was 10.6%, corresponding to approximately 480 million cases worldwide [2]. In Poland, data published by the Ministry of Health in the Maps of Health Needs indicated that in 2018, morbidity due to COPD was recorded in more than 1.2 million patients. In the same year, over 70,000 deaths were attributed to this disease [3].

A major challenge in the comprehensive management of COPD is the presence of comorbidities. Patients with COPD are more likely to develop concomitant diseases than those without COPD [4]. Evidence indicates that more than 30% of patients with COPD have at least one comorbid condition, and an additional 40% have two or more, with prevalence increasing with age. It is important to emphasize that comorbidities significantly and adversely influence the course of COPD [5,6,7,8,9,10].

Exacerbations are a component of the natural history of COPD and have a detrimental impact on long-term outcomes. According to the GOLD 2024 definition [1], a COPD exacerbation is *an acute event with symptoms worsening over a few days (up to 14 days) and characterized by increased dyspnea and/or cough and sputum that may be accompanied by tachypnea and/or tachycardia and is often associated with increased local and systemic inflammation caused by infection, pollution, or other insult to the airways*. In addition to symptom control, prevention of exacerbations is a primary therapeutic goal in COPD management [1].

Exacerbations are a frequent cause of hospitalization in patients with COPD. In Poland, the 30-day rehospitalization rate in this group exceeds 9%, and mortality within 90 days after discharge is greater than 10% [11]. These relatively high rates provide a compelling rationale for improving not only inpatient care standards but also post-discharge management.

A fundamental element of the hospital discharge process is the issuance of recommendations for further care. These are included in the hospital discharge summary, which serves not only as a permanent record of the hospitalization but also as the principal means of communication between inpatient and outpatient care. In this context, effective communication is essential [12,13,14,15]. To optimize this process, a panel of Polish experts developed the recommendations presented in this document regarding the content of discharge instructions following hospitalization for COPD exacerbation.

## 2. Methods

To establish a consensus based on the opinions of independent experts, a modified Delphi method was adopted.

At the initial stage of guideline development, an expert panel was convened, comprising seven specialists in pulmonary medicine (all members of the Polish Respiratory Society) and one specialist in family medicine. Panel members were selected based on their previous publications and extensive clinical experience in the management of COPD. Together, the panel represented key domains of expertise essential for the development of this document, including pharmacological and non-pharmacological treatment of COPD, management of respiratory failure, pulmonary rehabilitation, research methodology, as well as health policy and healthcare organization. The group included physicians with experience in inpatient care, outpatient specialist care, and primary healthcare.

All invited experts agreed to participate in the project. Subsequently, three panel discussions were held to identify and refine key issues requiring further elaboration. These issues were considered in the context of an international literature review, while also taking into account local determinants related to the specific characteristics of the Polish healthcare system. As a result of these discussions, a final list of issues was generated for voting. A project coordinator (A.J.B.) was appointed from within the expert panel.

In the subsequent stage, experts were asked to express their opinion on each issue using an 11-point Likert scale ranging from −5 (strongly disagree) to +5 (strongly agree). Consensus was defined a priori as a mean Likert score of ≥2.5 or ≤−2.5, corresponding to consensus “in favor of” or “against” a given statement, provided that the standard deviation did not cross zero. Scores between −2.5 and +2.5 indicated a lack of consensus. Voting was conducted via email. Each questionnaire allowed experts to propose modifications, which were then subjected to voting in subsequent rounds.

It was assumed that the recommendations presented in this document may be revised and updated in the event of newly published significant research findings or substantial changes in healthcare policy in Poland that could materially affect the content of the present statement.

The recommendations are summarized in Table 1.

## 3. Content of Recommendations

**Recommendation 1:**

**We recommend that the overall layout and formatting of the discharge summary be carefully designed to ensure a high level of readability for both healthcare professionals and patients.**

**Voting outcome:** mean score: 4.63

**Result:** Consensus in favor of the recommendation.

**Comment:** Ensuring adequate readability of the section containing discharge recommendations for the patient is particularly important given the general characteristics of individuals with COPD (older age and frequent comorbidities, including cognitive impairment) [1]. Based on their clinical experience, the experts noted that discharge summaries following hospitalization for COPD exacerbation are often not read carefully by patients. Potential reasons include excessive length, poor organization, the presence of numerous recommendations, and a lack of specific guidance on how to implement them in practice. Therefore, it is essential to maintain clarity in discharge documents to highlight key information. This will facilitate an effective and safe transition of care from the inpatient to the outpatient setting [16].

It is also crucial to clearly separate discharge recommendations intended for the patient from those directed to the physician providing follow-up care. A distinct section with patient-specific recommendations should therefore be created [17], and this approach should be implemented within hospital electronic health record templates.

The following measures are recommended to improve the readability of the discharge summary for both physicians and patients:

- Ensure conciseness, avoiding excessively long and detailed texts [16,18].
- Avoid large blocks of uninterrupted text [18].
- Structure content into thematic paragraphs with appropriate headings [18,19].
- Break written instructions into smaller, concrete steps [20].
- Avoid medical jargon and unexplained abbreviations [16,17].
- Use a larger font size for printed materials (minimum size 12) [19].

These aspects were also considered in the development of educational materials for patients, which are attached to this statement (see Supplementary S1–S3).

**Figure 1:**
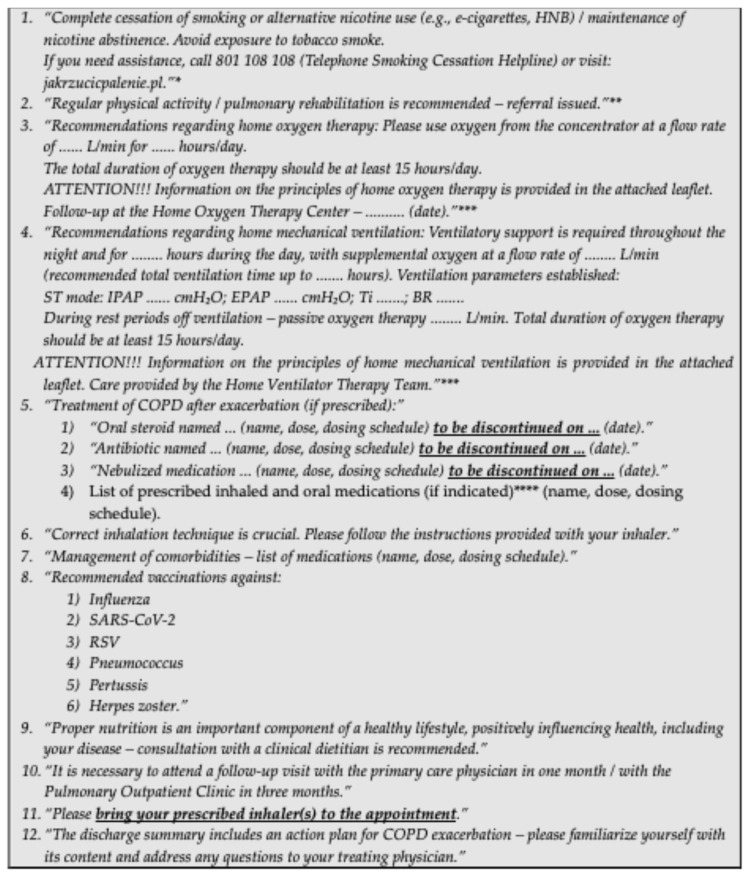
Proposal of concise discharge recommendations to be included in the information sheet for patients discharged after hospitalization for COPD exacerbation. **Notes:** * applies to smoking patients; ** after consideration of potential contraindications; *** applies to patients with respiratory failure; **** according to current knowledge, the lowest possible number of inhalers should be used, with preference for combination medications; if two inhalers are prescribed, they should be of the same type. **Abbreviations:** BR—Backup Rate; EPAP—Expiratory Positive Airway Pressure; HNB—Heat-Not-Burn; IPAP—Inspiratory Positive Airway Pressure; RSV—Respiratory Syncytial Virus; SARS-CoV-2—Severe Acute Respiratory Syndrome Coronavirus 2; Ti—Inspiratory Time; ST mode—Spontaneous/Timed mode.

A proposal of concise discharge recommendations for patients is presented in Figure 1.

**Recommendation 2:**

**We recommend that the preparation of the discharge summary involve a multidisciplinary team (physician, nurse, physiotherapist, and, optimally, also a dietitian and clinical psychologist). This will allow integration of discharge documents prepared by individual team members into a coherent whole.**

**Voting outcome:** mean score: 4.0

**Result:** Consensus in favor of the recommendation.

**Comment:** Experts recommend that discharge summaries be prepared by a multidisciplinary team [21,22] (in the Polish healthcare setting, optimally including a physician, nurse, physiotherapist, dietitian, and clinical psychologist). In addition to the physician, each specialist should contribute to developing detailed discharge recommendations within their area of competence.

Experts emphasized the importance of implementing a structured process for preparing discharge recommendations. A common practice is the use of pre-prepared templates, and the multidisciplinary team should be involved in their creation as well as in regular updates to ensure alignment with the current medical knowledge.

Although the physician remains the primary coordinator of the discharge process, active participation of other healthcare professionals is recommended to provide detailed, patient-specific recommendations according to their respective expertise. At the stage of integrating contributions from different specialists, consistency must be verified and any discrepancies resolved before the discharge summary is issued. Delivering inconsistent, or especially contradictory, recommendations can negatively affect the health literacy of patients with pulmonary diseases [23].

**Recommendation 3:**

**We recommend that discharge recommendations be discussed with the patient during the hospital stay.**

**Voting outcome:** mean score: 4.5

**Result:** Consensus in favor of the recommendation.

**Comment:** During hospitalization for COPD exacerbation, patients consistently report the need for better communication regarding post-discharge care [24,25]. Discharge recommendations should therefore be reviewed in person, giving patients the opportunity to ask questions and receive clarifications. Highlighting key information with a marker during this discussion can further improve understanding.

Whenever possible, caregivers should also be included in the education process, as they may play a crucial role in supporting comprehension and adherence [26,27,28,29].

**Recommendation 4:**

**We recommend that discharge recommendations be supplemented with additional educational materials provided independently of the discharge summary.**

**Voting outcome:** mean score: 4.13

**Result:** Consensus in favor of the recommendation.

**Comment:** A balance between the conciseness required for presenting the key discharge recommendations and the detail necessary for comprehensive information can be achieved by providing patients with additional educational materials. Evidence shows that educational leaflets, even when used as the sole intervention, positively impact knowledge among patients with COPD [30,31].

**Recommendation 5:**

**We recommend that discharge recommendations include instructions on the correct inhaler technique. A standardized template dedicated to a specific inhaler should be used as an additional educational material provided independently of the discharge summary.**

**Voting outcome:** mean score: 4.63

**Result:** Consensus in favor of the recommendation.

**Comment:** Incorrect inhalation technique has been consistently observed for decades [32]. Nevertheless, recommendations on proper inhaler use are rarely included in discharge summaries [33]. A key component of such recommendations is instructing patients to carefully review the user manual of the prescribed inhaler. However, as emphasized in the GOLD report, this measure alone may be insufficient [1]. Educational videos can serve as valuable support. It is also important to inform patients that incorrect technique reduces treatment effectiveness [34]. If a spacer device is prescribed, this should be explicitly noted in the discharge summary.

**Recommendation 6:**

**We recommend that discharge recommendations for patients using tobacco or nicotine products include information on the necessity of and strategies for cessation. A standardized template should also be used as an additional educational material provided independently of the discharge summary.**

**Voting outcome:** mean score: 5.0

**Result:** Consensus in favor of the recommendation.

**Comment:** The GOLD guidelines recommend providing patients with additional materials on smoking cessation [1]. Even in the absence of other forms of support, such an intervention appears effective [35]. Nevertheless, smoking cessation guidance is rarely included in discharge summaries [33]. This is concerning, as tobacco smoking remains the main environmental risk factor for COPD, and its cessation is essential to slowing disease progression [1].

**Recommendation 7:**

**We recommend that discharge recommendations include information on physical activity and pulmonary rehabilitation. A standardized template should be used as an additional educational material provided independently of the discharge summary.** [Supplementary S1].

**Voting outcome:** mean score: 4.88

**Result:** Consensus in favor of the recommendation.

**Comment:** Physical activity is a key component of COPD management, as it reduces both the risk of exacerbations and mortality [36]. Each patient should be assessed for possible contraindications to exercise. Recommendations on physical activity should cover both aerobic (endurance) and muscle-strengthening exercises, specifying frequency, intensity, time, and type of activity (FITT) [37].

Patients recovering from an exacerbation requiring hospitalization should also be referred to pulmonary rehabilitation [1,38]. Nevertheless, discharge recommendations on physical activity are rarely included in discharge summaries, and referrals for pulmonary rehabilitation are issued to only a small proportion of patients [11,33].

**Recommendation 8:**

**We recommend that discharge recommendations include a referral for dietary counseling.**

**Voting outcome:** mean score: 4.25

**Result:** Consensus in favor of the recommendation.

**Comment:** Malnutrition, due to its negative impact on the clinical course and natural history of COPD, is a significant problem in this patient population [39,40,41]. Therefore, referral for dietary counseling should be included in the discharge recommendations.

**Recommendation 9:**

**We recommend that discharge recommendations include information on vaccinations. A standardized template should also be used as an additional educational material provided independently of the discharge summary.** [Supplementary S2].

**Voting outcome:** mean score: 5.0

**Result:** Consensus in favor of the recommendation.

**Comment:** Prevention of respiratory tract infections is a key factor in reducing exacerbations and lowering mortality among patients with COPD. The effectiveness of pneumococcal and influenza vaccination is best documented in this regard [42,43,44,45]. However, vaccination coverage against these pathogens remains insufficient in this patient group [46]. Notably, influenza vaccination rates among Polish patients are among the lowest in Europe [47]. The most frequently reported reason for non-vaccination is the absence of a physician’s recommendation [46].

In addition, vaccination against SARS-CoV-2, herpes zoster (for patients over 50 years of age), and pertussis is also recommended [1].

**Recommendation 10:**

**We recommend that discharge recommendations include an action plan for COPD exacerbation. A standardized template of such a plan should be provided independently of the discharge summary** [Supplementary S3].

**Voting outcome:** mean score: 4.63

**Result:** Consensus in favor of the recommendation.

**Comment:** A COPD Action Plan is a set of instructions outlining the steps a patient should take in response to changes in symptom severity or nature that may indicate an exacerbation [1,48]. Given patients’ reported need to strengthen their competence in this area [24,49], such a plan should be included in the discharge recommendations. This is further justified by the high risk of rehospitalization following a COPD exacerbation. Moreover, many patients delay seeking medical attention despite experiencing worsening symptoms.

**Recommendation 11:**

**We recommend that discharge recommendations emphasize any changes to baseline COPD therapy and management of comorbidities.**

**Voting outcome:** mean score: 4.5

**Result:** Consensus in favor of the recommendation.

**Comment:** Exacerbations of COPD may necessitate modifications of maintenance therapy [1]. After an exacerbation, patients should receive a medication list that distinguishes drugs prescribed for COPD from those prescribed for comorbidities. This list should specify drug names, dosages, administration schedules, and, where applicable, planned discontinuation dates. This is particularly important for systemic steroids, as prolonged use may cause serious complications [50]. Patients should also be clearly informed that therapy must not be modified independently, including resuming medications or regimens used before the exacerbation.

**Recommendation 12:**

**We recommend that patients with COPD and concomitant respiratory failure receive discharge recommendations regarding home oxygen therapy or home mechanical ventilation. A standardized template should be used as an additional educational material provided independently of the discharge summary.**

**Voting outcome:** mean score: 4.75

**Result:** Consensus in favor of the recommendation.

**Comment:** Respiratory failure is a frequent complication of COPD [51]. Patients meeting the appropriate criteria should receive long-term oxygen therapy (LTOT) at home [52]. Hospitalization for exacerbation requires reevaluation of ongoing oxygen therapy and the inclusion of updated recommendations in the discharge summary. Hospitalization is also often the point at which respiratory failure is diagnosed for the first time. The need for further LTOT should be reassessed in a specialized Home Oxygen Therapy Center three months after discharge, during a stable disease period, by performing arterial blood gas analysis [53].

A subgroup of COPD patients also requires home mechanical ventilation [1]. These patients should receive discharge recommendations covering all aspects of ventilation, including duration, parameters, rest therapy, and information on care provided by the Home Ventilator Therapy Team (DLR). A proposal of concise recommendations for inclusion in the patient information sheet is presented in Figure 1.

**Recommendation 13:**

**We recommend that discharge recommendations include information on the necessity of bringing inhaler(s) to the follow-up visit.**

**Voting outcome:** mean score: 4.63

**Result:** Consensus in favor of the recommendation.

**Comment:**

Regular verification of correct inhaler use is an essential component of the COPD therapeutic cycle [1]. Therefore, instructions to bring inhalers to the follow-up visit should be included as an integral part of the discharge recommendations.

**Recommendation 14:**

**We recommend that the discharge summary include information on the necessity of attending a follow-up visit.**

**Voting outcome:** mean score: 4.63

**Result:** Consensus in favor of the recommendation

**Comment:** Patients should attend a follow-up visit within four weeks after hospital discharge [1]. This approach appears to reduce the risk of rehospitalization [54,55]. Depending on the scope, the visit may take place either in primary care or in specialist outpatient care.

The follow-up visit should include an assessment of the patient’s understanding of the treatment regimen and adherence to discharge recommendations [1]. The following elements are recommended:

1. Review of the discharge summary.
2. Documentation of the exacerbation (medical records/individual medical care plan)
3. Discussion with the patient regarding the reason for hospitalization and further diagnostic and therapeutic management.
4. Assessment of symptom severity (mMRC [modified Medical Research Council]/COPD Assessment Test [CAT]).
5. Evaluation of physical activity.
6. Verification of vaccination status and discussion of recommended vaccinations
7. Assessment of comorbidities:
  - Indications for further diagnostic evaluation;
  - Evaluation of control of established comorbidities;
  - Assessment of cardiovascular risk.
8. Review of current medications, with attention to modifications introduced during hospitalization, including tapering schedules for medications used in the exacerbation (e.g., oral corticosteroids, antibiotics).
9. Assessment of inhaler technique.
10. Measurement of oxygen saturation using pulse oximetry.
11. Smoking cessation intervention for active smokers.
12. Patient education in areas of care identified as important, including dietary counseling.
13. Modification or issuance of a written action plan.
14. Planning of the next follow-up visit and diagnostic tests (including evaluation of indications for a pulmonology consultation).

## 4. Conclusions

The PRS expert working group has developed recommendations on the content of discharge instructions following hospitalization for COPD exacerbation. The experts emphasize the need for a comprehensive approach to the discharge process. Key elements include careful preparation of the discharge summary to ensure readability for both physicians and patients, and the involvement of a multidisciplinary team in its development.

Furthermore, the experts highlight the importance of discussing discharge recommendations with the patient during hospitalization and advise supplementing them with educational materials conveying key areas such as inhaler technique, smoking cessation, physical activity, preventive vaccinations, management of respiratory failure, modifications to maintenance therapy, and an action plan for COPD exacerbation.

The discharge summary should also include information on the necessity of attending a follow-up visit and bringing prescribed inhalers to the appointment. Implementation of these recommendations is expected to improve the quality of care for patients with COPD after hospitalization for an exacerbation.

## Figures and Tables

**Table 1:** Summary of the Polish Respiratory Society expert recommendations on the content of discharge instructions following hospitalization for COPD exacerbation.

No.	Recommendation	Voting Outcome
1	We recommend that the overall layout and formatting of the discharge summary be carefully designed to ensure a high level of readability for both healthcare professionals and patients.	Consensus in favor
2	We recommend that the preparation of the discharge summary involve a multidisciplinary team (physician, nurse, physiotherapist, and, optimally, also a dietitian and clinical psychologist). This will allow integration of discharge documents prepared by individual team members into a coherent whole.	Consensus in favor
3	We recommend that discharge recommendations be discussed with the patient during the hospital stay.	Consensus in favor
4	We recommend that discharge recommendations be supplemented with additional educational materials provided independently of the discharge summary.	Consensus in favor
5	We recommend that discharge recommendations include instructions on the correct inhaler technique. A standardized template dedicated to a specific inhaler should be used as an additional educational material provided independently of the discharge summary.	Consensus in favor
6	We recommend that discharge recommendations for patients using tobacco or nicotine products include information on the necessity of and strategies for cessation. A standardized template should also be used as an additional educational material provided independently of the discharge summary.	Consensus in favor
7	We recommend that discharge recommendations include information on physical activity and pulmonary rehabilitation. A standardized template should be used as an additional educational material provided independently of the discharge summary.	Consensus in favor
8	We recommend that discharge recommendations include a referral for dietary counseling.	Consensus in favor
9	We recommend that discharge recommendations include information on vaccinations. A standardized template should also be used as an additional educational material provided independently of the discharge summary.	Consensus in favor
10	We recommend that discharge recommendations include an action plan for COPD exacerbation. A standardized template of such a plan should be provided independently of the discharge summary.	Consensus in favor
11	We recommend that discharge recommendations emphasize any changes to baseline COPD therapy and management of comorbidities.	Consensus in favor
12	We recommend that patients with COPD and concomitant respiratory failure receive discharge recommendations regarding home oxygen therapy or home mechanical ventilation. A standardized template should be used as an additional educational material provided independently of the discharge summary.	Consensus in favor
13	We recommend that discharge recommendations include information on the necessity of bringing inhaler(s) to the follow-up visit.	Consensus in favor
14	We recommend that the discharge summary include information on the necessity of attending a follow-up visit.	Consensus in favor

## Data Availability

Not applicable.

## Supplementary Materials

The following supporting information can be downloaded at: https://www.mdpi.com/article/10.3390/arm94010004/s1, Supplementary S1—Recommendations on physical activity for patients with COPD; Supplementary S2—Recommendations on vaccinations for patients with COPD; Supplementary S3—COPD action plan.

## Abbreviations

The following abbreviations are used in this manuscript:

BR	Backup Rate
COPD	Chronic Obstructive Pulmonary Disease
EPAP	Expiratory Positive Airway Pressure
GOLD	Global Initiative for Chronic Obstructive Lung Disease
HNB	Heat-Not-Burn
IPAP	Inspiratory Positive Airway Pressure
PRS	Polish Respiratory Society
RSV	Respiratory Syncytial Virus
SARS-CoV-2	Severe Acute Respiratory Syndrome Coronavirus 2
ST mode	Spontaneous/Timed mode
Ti	Inspiratory Time
