# Supplementary material for: Recommendations Following Hospitalization for Acute Exacerbation of COPD—A Consensus Statement of the Polish Respiratory Society
Source: Adv Respir Med. 2026 Jan 4;94(1):4. doi: 10.3390/arm94010004 (PMC12821542; doi:10.3390/arm94010004)
Supplement: Supplementary file 1 [file arm-94-00004-s001.zip › Supplementary S1.pdf]

Recommendations on Physical Activity for Patients with COPD

Aerobic exercise:

Moderate intensity, at least **150 minutes per week** (ideally 30–60 minutes, 5 times a week). You may exercise in one longer session or in shorter bouts of at least 10 minutes.

If you have not been physically active so far, start with **10-minute sessions** and add 5 minutes to each session every 2–4 weeks, until you reach the desired 30 minutes.

Preferred activities: brisk walking, Nordic walking, swimming, cycling.

Exercise intensity will be set individually - based on an exercise test or on how hard the exercise feels to you (The Rate of Perceived Exertion Scale - RPE). When using RPE, try to aim for a level of 4–6.

The RPE scale:

Rating	Perceived Exertion Level
**0**	No exertion (at rest)
**1**	Very light
**2**	Light
**3**	
**4**	Moderate (somewhat hard)
**5**	
**6**	High (vigorous)
**7**	
**8**	Very hard
**9**	
**10**	Maximum effort (highest possible)

Breathing technique during exercise: Your doctor or physiotherapist will teach you the so-called *pursed-lip breathing* technique or how to use specific devices.

Pursed-lip breathing:

1. **Breathe in slowly through your nose for about 2 seconds,** keeping your mouth closed. You should feel your belly gently rise as you inhale. Some people find it helpful to place a hand on their abdomen.
2. Gently tighten your lips, leaving a small opening to create slight resistance to airflow during exhalation.
3. Breathe out slowly and gently through your *pursed lips* for four seconds or longer. Exhale normally. Do not force the air out of your lungs; breathe out longer than you breathe in, and maintain a slow, relaxed breathing pattern until you feel fully in control of your breathing.

<u>Always follow the recommendations and safety rules discussed with your doctor/physiotherapist at discharge.</u> In particular, please remember:

- If you use an oxygen concentrator – your doctor will also explain how to use it during exercise.
- Drink plenty of water before, during, and after exercise.
- If you exercise outdoors – avoid training in cold weather or in uncertain weather conditions.

Strength training:

At least **twice a week** (note: do not train the same muscle group two days in a row – ask your doctor/physiotherapist for details), working on each major muscle group.

All recommended exercises will be included in your individual training plan.

The following terms explain basic words used in exercise instructions:

Repetition – one full movement of an exercise.

Set – a group of repetitions performed one after another without resting.

<u>ATTENTION!!! Do not hold your breath while exercising!</u>

Exercises are best performed under the supervision of a qualified trainer or physiotherapist. **<u>Please inform your primary care doctor or pulmonologist about any difficulties in exercising.</u>**

INDIVIDUAL TRAINING PLAN

PATIENT NAME: ____
DATE: ___/___/___

Aerobic training:

Type	Frequency	Intesity	Time	Comments

Strength training:

	Exercise	Weight [kg]	Number of repetitions	Number of sets	Rest [min]	Comments
1						
2						
3						
4						
5						
6						
7						
8						
9						
10						

Additional Comments:

Source: Recommendations Following Hospitalization for Acute Exacerbation of COPD – A Consensus Statement of the Polish Respiratory Society
