# Supplementary material for: Recommendations Following Hospitalization for Acute Exacerbation of COPD—A Consensus Statement of the Polish Respiratory Society
Source: Adv Respir Med. 2026 Jan 4;94(1):4. doi: 10.3390/arm94010004 (PMC12821542; doi:10.3390/arm94010004)
Supplement: Supplementary file 1 [file arm-94-00004-s001.zip › Supplementary S2.pdf]

Recommendations on Preventive Vaccinations for Patients with COPD

Recommendation for Patient
**Influenza vaccination**<br>Annual influenza vaccination is recommended<br>The optimal time for influenza vaccination is between September and December<br><br>**Recommended date of the next vaccination: …**
**Pneumococcal vaccination**<br>1 dose of conjugate vaccine PCV20<br>or<br>1 dose of conjugate vaccine PCV13 followed by 1 dose of polysaccharide vaccine PPSV23, with an interval of at least 8 weeks between the two<br><br>**Recommended date of vaccination: …**
**COVID-19 vaccination**<br><br>**Recommended date of vaccination: …**
**Pertussis vaccination**<br><br>**Recommended date of vaccination: …**
**Herpes zoster (shingles) vaccination**<br>2 doses of vaccine, administered 2 months apart<br><br>**Recommended date of vaccination: …**
**Respiratory syncytial virus (RSV) vaccination**<br>1 dose of vaccine<br><br>**Recommended date of vaccination: …**

Source: Recommendations Following Hospitalization for Acute Exacerbation of COPD – A Consensus Statement of the Polish Respiratory Society
