# Supplementary material for: Recommendations Following Hospitalization for Acute Exacerbation of COPD—A Consensus Statement of the Polish Respiratory Society
Source: Adv Respir Med. 2026 Jan 4;94(1):4. doi: 10.3390/arm94010004 (PMC12821542; doi:10.3390/arm94010004)
Supplement: Supplementary file 1 [file arm-94-00004-s001.zip › Supplementary S3.pdf]

REMEMBER!

The most common reason for ineffective COPD treatment or worsening of symptoms is **not following medical recommendations** – in particular, irregular use of medications, incorrect inhaler technique, or stopping medications altogether.

This action plan has been created to make COPD management easier for you – both in everyday life and in case of worsening symptoms.

Read this plan carefully and refer back to it whenever you have doubts.

If you have any further questions – ask your doctor.

The most important factor causing COPD and worsening the health of patients who already suffer from the disease is tobacco smoke – whether from cigarettes, pipes, or other forms of smoking, as well as marijuana smoking.

What worsens my COPD?

To better understand your disease and prevent its exacerbations, it is important to know the factors that may trigger COPD flare-ups. These include:

- **AIR POLLUTION**
- **RESPIRATORY INFECTIONS**
- **TOBACCO SMOKE** – both when you smoke cigarettes or other tobacco products, and when you inhale tobacco smoke passively!

You should tell every doctor you visit that you have COPD!

REMEMBER!

It is extremely important that your primary care physician or pulmonologist knows about **EVERY ONE OF YOUR EXACERBATIONS**. Your treatment depends on this! Inform your doctor about any worsening of your health condition related to COPD.

Always remember to inform every doctor treating you that you have COPD!

Source: Recommendations Following Hospitalization for Acute Exacerbation of COPD – A Consensus Statement of the Polish Respiratory Society.

ACTION PLAN for a patient with COPD

My name and surname:

My lung doctor:

My primary care physician:

My primary care clinic phone number:

In case of emergency, notify:

My constant saturation level (%):

IT IS AS USUAL...

› I cough up the same amount and color of sputum as usual

› I can perform the same physical activities and daily household duties as usual

MY REGULAR COPD MEDICATIONS:

______

I take ___ inhalation(s) ___ times per day

______

I take ___ inhalation(s) ___ times per day

______

I take ___ inhalation(s) ___ times per day

MY RESCUE MEDICATION (TO BE USED IN CASE OF SYMPTOMS) IS:

______

I take ___ inhalation(s) when:

› I cough

› I have difficulty breathing

› My breathing is wheezy

› I feel short of breath / lack air / feel heaviness in my chest

OTHER TREATMENTS I USE FOR COPD:

I take ___ inhalation(s) ___ times per day

I use oxygen ___ hours per day, with a flow of ___ L/min

I DO NOT FEEL WELL...

› My measured oxygen saturation level is **3 percentage points (or more) lower** than my usual level

› I cough up **more sputum** than usual

› I cough up **purulent (yellow) sputum**

› I experience **more shortness of breath** than usual

› I need to **use my rescue inhaler more** often than usual

› I feel **tired**

› Daily physical activity and carrying out everyday household duties are **difficult for me**

WHAT SHOULD I DO?

1. If I have not done this before – I start using my regular maintenance medication consistently.

2. I use my rescue medication ___ inhalations every hour (for 2-3 doses), and then ___ inhalations every ___ hours.

› If I do not feel any improvement within 48 hours – I contact my primary care physician.

› If I cannot arrange an urgent appointment with my primary care physician, I go to the Out-of-Hours Medical Service or to the Emergency Department.

3. If my doctor prescribed an emergency oral steroid:

______

I take ___ mg immediately, and then for ___ days

Date of starting steroid therapy: ______

ATTENTION!!! I contact my primary care physician to inform them that I have started taking the steroid and I make a medical appointment.

If my doctor prescribed an emergency oral steroid,

______

I take: ___ mg ___ times per day for ___ days

DANGER!

I am feeling worse despite increased COPD treatment, including a higher dose of rescue medication, oral steroid, or antibiotic.

112

After taking the medications, contact your primary care physician.

If symptoms do not subside within 15 minutes, and you cannot reach your doctor or the doctor is unavailable – CALL 112!

ATTENTION! If:

› I am **confused or drowsy** despite oxygen therapy (if I feel this way – **I do not increase oxygen flow!**)

› Shortness of breath started **suddenly**

› To breathe better, I have to move my nostrils and/or my ribs are visible when I breathe

› Due to shortness of breath, **I have difficulty speaking or eating**

› The medications I have taken have **not helped**

› My lips and/or fingernails are **bluish or gray**

› **I feel anxious**

› I feel **chest pain**

112

IMMEDIATELY AFTER TAKING RESCUE MEDICATION INHALATIONS 112!

Continue using your rescue medication until the Emergency Medical Team arrives.

Apply breathing control techniques as previously instructed.
